# Identification of improved IL28B SNPs and haplotypes for prediction of drug response in treatment of hepatitis C using massively parallel sequencing in a cross-sectional European cohort

**DOI:** 10.1186/gm273

**Published:** 2011-08-31

**Authors:** Katherine R Smith, Vijayaprakash Suppiah, Kate O'Connor, Thomas Berg, Martin Weltman, Maria Lorena Abate, Ulrich Spengler, Margaret Bassendine, Gail Matthews, William L Irving, Elizabeth Powell, Stephen Riordan, Golo Ahlenstiel, Graeme J Stewart, Melanie Bahlo, Jacob George, David R Booth

**Affiliations:** 1 Bioinformatics Division, The Walter and Eliza Hall Institute of Medical Research, 1G Royal Parade, Parkville, Victoria 3052, Australia; 2 Storr Liver Unit, Westmead Millennium Institute, University of Sydney, Sydney, NSW 2145, Australia; 3 Institute for Immunology and Allergy Research, Westmead Millennium Institute, University of Sydney, Sydney, NSW 2145, Australia; 4 Medizinische Klinik m.S. Hepatologie und Gastroenterologie, Charité, Campus Virchow-Klinikum, Universitätsmedizin Berlin; 5 Department of Hepatology, Clinic for Gastroenterology and Rheumatology, University Clinic Leipzig, Leipzig, 04103, Germany; 6 Department of Gastroenterology and Hepatology, Nepean Hospital, Sydney, NSW, 2145, Australia; 7 Liver Physiopathology Lab, Department of Internal Medicine, University of Turin, Turin, 10060, Italy; 8 Department of Internal Medicine I, University of Bonn, Sigmund-Freud-Strasse 25, Bonn, 53127, Germany; 9 Liver Research Group, Institute of Cellular Medicine, Medical School, Newcastle University, Newcastle upon Tyne, NE2 4HH, UK; 10 National Centre in HIV Epidemiology and Clinical Research, University of New South Wales, Sydney, Australia; 11 St Vincent's Hospital, Sydney, NSW 2010, Australia; 12 NIHR Biomedical Research Unit in Gastroenterology and the Liver, University of Nottingham, Nottingham, NG7 2UH, UK; 13 Princess Alexandra Hospital, Department of Gastroenterology and Hepatology, Ipswich Road, Woolloongabba, Queensland 4102, Australia; 14 The University of Queensland, School of Medicine, Princess Alexandra Hospital, Ipswich Road, Woolloongabba, Queensland 4102, Australia; 15 Gastrointestinal and Liver Unit, Prince of Wales Hospital and University of New South Wales, Sydney, NSW 2010, Australia; 16 Department of Mathematics and Statistics, The University of Melbourne, Victoria 3010, Australia

## Abstract

**Background:**

The hepatitis C virus (HCV) infects nearly 3% of the World's population, causing severe liver disease in many. Standard of care therapy is currently pegylated interferon alpha and ribavirin (PegIFN/R), which is effective in less than half of those infected with the most common viral genotype. Two *IL28B *single nucleotide polymorphisms (SNPs), rs8099917 and rs12979860, predict response to (PegIFN/R) therapy in treatment of HCV infection. These SNPs were identified in genome wide analyses using Illumina genotyping chips. In people of European ancestry, there are 6 common (more than 1%) haplotypes for *IL28B*, one tagged by the rs8099917 minor allele, four tagged by rs12979860.

**Methods:**

We used massively parallel sequencing of the *IL28B *and *IL28A *gene regions generated by polymerase chain reaction (PCR) from pooled DNA samples from 100 responders and 99 non-responders to therapy, to identify common variants. Variants that had high odds ratios and were validated were then genotyped in a cohort of 905 responders and non-responders. Their predictive power was assessed, alone and in combination with HLA-C.

**Results:**

Only SNPs in the *IL28B *linkage disequilibrium block predicted drug response. Eighteen SNPs were identified with evidence for association with drug response, and with a high degree of confidence in the sequence call. We found that two SNPs, rs4803221 (homozygote minor allele positive predictive value (PPV) of 77%) and rs7248668 (PPV 78%), predicted failure to respond better than the current best, rs8099917 (PPV 73%) and rs12979860 (PPV 68%) in this cross-sectional cohort. The best SNPs tagged a single common haplotype, haplotype 2. Genotypes predicted lack of response better than alleles. However, combination of *IL28B *haplotype 2 carrier status with the HLA-C C2C2 genotype, which has previously been reported to improve prediction in combination with *IL28B*, provides the highest PPV (80%). The haplotypes present alternative putative transcription factor binding and methylation sites.

**Conclusions:**

Massively parallel sequencing allowed identification and comparison of the best common SNPs for identifying treatment failure in therapy for HCV. SNPs tagging a single haplotype have the highest PPV, especially in combination with HLA-C. The functional basis for the association may be due to altered regulation of the gene. These approaches have utility in improving diagnostic testing and identifying causal haplotypes or SNPs.

## Background

Some 3% of the World's population is infected with the hepatitis C virus (HCV). In most cases, the virus, if either untreated or treated but not cleared, causes chronic infection and thereby increases the risk of liver failure and liver cancer. HCV infection is also the major cause of liver transplantation. It is consequently desirable to eradicate the virus before end stage liver disease develops and to improve transplant outcomes by preventing reinfection in the transplanted liver. The current standard of care is pegylated interferon and ribavirin, which clears the virus only after weekly injections for up to 48 weeks and in less than half of those infected with the most common form of the virus, genotype 1.

In 2009, four independent groups, using genome wide association studies (GWAS), identified SNPs from the *IL28B *(RefSeq accession [RefSeq:NM_172139]) region that could predict drug response [1-4]. A further study demonstrated that viral clearance without therapy was also predicted by these SNPs [5]. Previously, prediction of treatment failure was based on phenotypic features such as viral load, body mass index, ethnicity, and liver fibrosis. *IL28B *genotype, however, predicts treatment failure with greater sensitivity and specificity. Subsequently, serum IP10 [6], 25 hydroxy vitamin D_3 _[7], and hepatic ISG expression from biopsies were also found to predict response [8]. Combinations of these factors, the *IL28B *genotype, and the HLA-C genotype [9] have proven effective in predicting therapeutic response.

It is desirable to predict treatment response for a number of reasons. For those unlikely to respond, alternative therapies are in late phase clinical trials and have a greatly improved success rate due to the ability to target those that are unlikely to respond to the standard treatment. Patients with non-response genotypes could therefore delay treatment, or have preferential use of the more expensive new therapies, which are all designed to be used in combination with the current standard of care. The predictive value of SNPs is best calculated from routine clinical practice, rather than the clinical trial scenario, since these are the conditions in which most patients are treated and where there is usually much lower compliance with drug usage regimens. Consequently, in this study we used a cross-sectional cohort to compare response rates for the different SNPs.

Other groups [1,2] used Sanger sequencing of the coding region of IL28B, or genotyping of previously identified SNPs from this region or the flanking regions [10,11] to search for SNPs not on the GWAS chips which might predict drug response with higher specificity or sensitivity. Here we describe our use of massively parallel sequencing (MPS) to detect SNPs or indels over the 100 kb around *IL28B*. MPS allows the high throughput detection of new and less common variants and is now a routine follow up for GWAS hits. MPS may identify further variants better able to predict drug response than those discovered through the GWAS SNP chip design, which usually only detects association by proxy, or indirect association, i.e. does not identify the causal variants. Thus, newly discovered variants could include the identification of additional haplotype tagging SNPs, SNPs that did not tag haplotypes, less common SNPs with better predictive values than the common haplotypes, and possibly synthetic SNPs that were tagged by the response haplotypes.

MPS is still an expensive technology. Thus the most suitable design for re-sequencing of a GWAS hit is currently via a pooling strategy. Such a strategy will only have limited power to identify rare variants.

### The *IL28B *region

**Figure 1:**
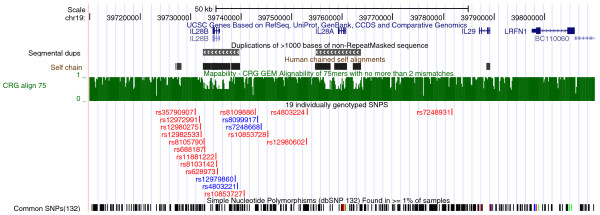
**UCSC screenshot of the chromosome 19 region containing *IL28A, IL28B *and *IL29***. Coordinates are from hg19. *IL28A *and *IL28B *lie within segmental duplications. The locations of these duplications are reflected in areas of poor mapability, as indicated by low scores on the CRG Align 75 subtrack. The score for this subtrack is the reciprocal of the number of matches found in the genome for 75 mers with no more than 2 mismatches. The track below this subtrack shows the location of the 19 SNPs that were individually genotyped using Sequenom. The four SNPs that best tagged the *IL28B *region haplotypes are indicated in blue. Screenshot taken from UCSC draft human genome [28].

*IL28B *lies next to two related genes, *IL28A *(NM_172138) and *IL29 *(NM_172140), on chromosome 19 (Figure 1, [12]). The three genes are thought to have evolved via two gene duplication events [13]. The resulting degree of homology makes sequencing and alignment in this region particularly challenging, with the problem particularly acute for *IL28A *and *IL28B*, which share identity for 1309 bases over a 1339 bp length. Anticipating this problem, we chose to perform paired-end sequencing, which allows a read pair to be unambiguously mapped if one end can be unambiguously mapped, regardless of whether the other end maps to multiple locations. However, due to the extended region of similarity, it remains possible that both reads of a pair may map to multiple locations.

## Methods

### Ethics statement and study subjects

Ethical approval was obtained from the Human Research Ethics Committees of Sydney West Area Health Service and the University of Sydney. All other sites had ethical approval from their respective ethics committees. Written informed consent was obtained from all participants. Characteristics of each cohort are shown in Table 1. All treated patients were infected with genotype 1, received pegylated interferon and ribavirin (PegIFN/R), and had virological response determined 6 months after completion of therapy. The diagnosis of chronic hepatitis C was based on appropriate serology and presence of HCV RNA in serum. All sustained virological responders (SVRs) and non-SVR cases received therapy for 48 weeks except when HCV RNA was present with a < 2 log drop in RNA level after 12 weeks therapy. Patients were classified as having had a sustained virological response (SVR) if they were HCV PCR negative, 6 months after the end of therapy. Patients were excluded if they had been co-infected with either hepatitis B virus (HBV) or human immunodeficiency virus (HIV) or if they were not of European descent.

**Table 1:** Demographic characteristics of chronic hepatitis C patients after therapy

	Demographic factors^a^				
		Gender			
	Mean years of age (SD)	Females	Males	Mean BMI (SD)	Viral load^c^
Australian cohort (*n *= 312,313)					
SVR (*n *= 130)	40.0 ^a ^(9.6)	52 ^b ^(40.0)	78 ^b ^(60.0)	26.927.0 (4.85.1)	*P *= NS
NSVR (*n *= 182,183)	44.5 4 ^a ^(7.12)	42 43 ^b ^(23.15)	140 ^b ^(76.95)	27.4 (5.32)	
					
Berlin cohort (*n *= 310,307)					
SVR (*n *= 150,149)	41.0 (10.5)	79 78 (52.73)	71 (47.37)	25.1 (4.5)	*P *< 0.05001
NSVR (*n *= 15,860)	46.7 8 (10.34)	69 68 (43.10)	91 90 (55.97.0)	25.9 (3.9)	
					
Newcastle UK cohort (*n *= 6,990)					
SVR (*n *= 3,140)	3837.2 ^a ^(11.86)	9 12 (29.030.0)	22 28 (7170.0)	25.23.7 (3.96.3)	*P *≤ 0.05002
NSVR (*n *= 5,038)	46.0 ^a ^(1210.0)	10 12 (26.324.0)	28 38 (73.776.0)	26.227.0 (6.64.8)	
					
Bonn cohort (*n *= 57)					
SVR (*n *= 26)	44.7 (12.9)	11 (42.3)	15 (57.7)	25.4 (4.23)	*P *≤ 0.05008
NSVR (*n *= 31)	50.8 (10.9)	11 (35.5)	20 (64.5)	27.3 (4.6)	
					
Trent UK cohort (*n *= 4,843)					
SVR (*n *= 2,221)	39.843.5 (9.80)	6 5 (27.323.8)	16 (72.776.2)	26.97.1 (3.57)	NS
NSVR (*n *= 2,622)	45.747.4 (7.99.3)	5 4 (1918.2)	21 18 (8081.8)	2526.0 (2.93.5)	
					
Turin cohort (*n *= 11,495)					
SVR (*n *= 5,845)	41.63.3 (13.10)	28 18 (48.340.0)	30 27 (51.760.0)	24.023.9 (3.23)	*P *≤ 0.0502
NSVR (*n *= 5,650)	45.1 7 (10.09.7)	19 (33.938.0)	37 31 (66.162.0)	24.5 6 (3.34)	
					
Total cohort (*n *= 910,905)					
SVR (*n *= 417,411)	40.9 6 ^a ^(10.87)	185 176 ^b ^(44.442.8)	232 235 ^b ^(55.657.2)	25.5 7 (4.75)	*P *< 0.05001
NSVR (*n *= 493,494)	45.7^b ^8 ^a ^(9.32)	156^b ^157 ^b ^(31.68)	337 ^b ^(68.42)	26.3 5 (4.76)	

^a^*P *< 0.001 comparisons between responders (SVR) and non-responders (NSVR) based on Student's t-testUnless otherwise specified, mean (s.d.) are presented. ^b^*P *< 0.05 005 comparisons between responders (SVR) and non-responders (NSVR) based on the χ^2 ^test. ^c^Comparisons between SVR and NSVR based on the Mann-Whitney U test.

### Massively parallel sequencing of DNA pools

**Figure 2:**
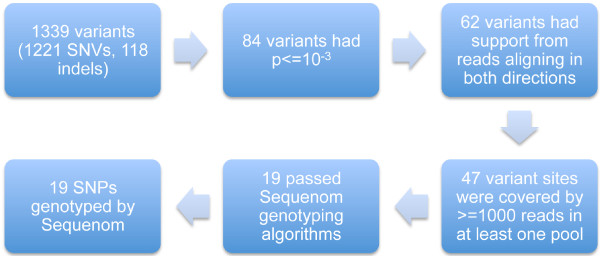
**SNP selection scheme**.

The study design is illustrated in Figure 2. DNA samples from 100 of the 131 responders studied by Suppiah et al were pooled to create a responder DNA pool, while DNA samples from 99 of the 162 non-responders were pooled to create a non-responder DNA pool. DNA samples were not barcoded.

Long-range PCR was used to amplify a continuous 100 kbp region of DNA containing the *IL28A, IL28B*, and *IL29 *genes. Specifically, 23 overlapping amplicons of size 4800-5273 bp were used to amplify chr19:39,709,944-39,809,945 (human genome version hg19). The PCR products from each pool were sequenced using a lane on an Illumina Genome Analyzer II flowcell, with 75 bp paired-end reads generated. Raw read quality was examined using FASTQC version 0.7.0 [14]. Genotype data has been deposited at the European Genome-phenome Archive (EGA) [15], which is hosted by the EBI, under accession number [EBI:EGAS00001000096].

### Alignment and post-processing

Reads from each pool were aligned separately to hg19 using version 0.5.8a of BWA [16]. Up to four mismatches were permitted across the length of each read, including up to four mismatches in the 32 bp seed. Bases with Phred quality scores of less than three were trimmed from the ends of reads.

Reads corresponding to the target region were extracted using SAMtools [17]. Reads with a mapping quality of zero were discarded. This discards reads mapped as singletons which do not align to unique location in the genome, as well as read pairs where neither end maps to a unique location.

Considering the small size of the target region, the large number of haplotypes present, and the depth of sequencing, we expected a substantial proportion of read pairs aligning to the same location to be biological rather than PCR duplicates. Hence, duplicate removal was not performed.

### Variant calling and association testing using pooled DNA

Variant calling was performed using version 3 of CRISP [18], a variant detection algorithm designed specifically for pooled DNA samples. Default settings were used (minimum read mapping/base quality to consider a read/base for variant calling 10; ≥ 1 read supporting variant must have mapping quality ≥ 20; ≥ 4 reads per pool; contingency table threshold p = 10^-3^; quality values based p-value threshold 10^-5^).

Fisher's exact test was used to compare the proportion of each allele in responders and non-responders at variant sites. If the total number of allele counts for a cohort exceeded the number of chromosomes, allele counts were rescaled so that they summed to the number of chromosomes in the cohort.

### Validation with original GWAS SNP data

15 SNPs within the 100 kbp target region were individually genotyped as part of the original GWAS. For these SNPs, we are able to compare the minor allele frequencies (MAFs), odds ratios and Fisher exact p-values estimated from pooled MPS data to those obtained from individual genotyping results. Correlation between the two sets of results was summarised using Spearman's rank correlation coefficient.

We also compared MAFs from pooled MPS to MAFs from CEPH genotypes, obtained from Utah residents with ancestry from northern and western Europe (CEU), for the 35 SNPs in the target region which were studied as part of the HapMap project [19].

### Validation using individual genotyping

19 putative single nucleotide variants (SNVs) were chosen for validation using individual genotyping based on the MPS results and/or biological grounds. We ranked the MPS SNPs by Fisher's exact test and rejected SNPs with p-values exceeding 10^-3^. Only SNPs supported by reads aligning in both directions and covered by at least 1000 reads in at least one pool were chosen. Of these 47 SNPs remaining we then excluded those failing Sequenom genotyping algorithms (excludes SNPs which are in highly homologous regions, have multiple genomic targets, or are close to other variants which may confound the genotyping). This left us with 19 SNPs, all included in dbSNP132. Ten had been genotyped in previous studies, and we genotyped the remainder in a single multiplex Sequenom reaction. The validation cohort comprised 905 samples from six different cohorts from Australia, the UK, Germany and Italy. 312 individuals were from our original GWAS (this includes the 199 patients who were in the R and NR pools), 581 from the replication phase, and 43 samples were not in the GWAS. Three SNPs (rs8105790, rs8103142 and rs628973) were not genotyped for 324 samples and 43 samples were not genotyped for five SNPs (rs10853727, rs8109886, rs10853728, rs12980602, rs4803224).

Genotype cleaning was performed using PLINK [20]. Accounting for obligate missingness, we discarded samples with a genotyping rate of below 89.47% (corresponding to less than 17/19 SNPs being assigned a genotype), and SNPs with (i) a genotyping rate below 90%, (ii) a minor allele frequency (MAF) below 1%, or (iii) a combined sample Hardy Weinberg test p-value below 10^-10^. We used Pearson's chi-square test to test for differential missingness of SNPs between responders and nonresponders.

Single marker association analyses were also performed using PLINK. We combined genotypes from the six cohorts and performed tests of association under allelic, genotypic, recessive and dominant genetic models. We also performed a fixed-effects meta-analysis for the allelic test; this was not deemed advisable under other genetic models as low or zero genotype counts resulted for some SNP/cohort combinations.

Linkage disequilibrium (LD) was estimated and haplotype frequencies inferred using Haploview [21]. The frequencies of common haplotypes (> = 1%) were compared between non-responders and responders using odds ratios, with 95% confidence intervals calculated using Woolf's formula and p-values calculated using Pearson's chi-square test.

## Results

### Massively parallel sequencing

The test and validation cohorts have been described previously, and their demographic features are summarised in Table 1. From the test cohort, the responder pool lane generated 34,026,026 reads (17,013,013 pairs) while the non-responder pool lane generated 34,236,380 reads (17,118,190 pairs). Inspection of raw reads using FASTQC analysis indicated some problems, in particular overrepresentation of particular sequences. These were discovered to correspond to the ends of amplicons, i.e. primers and adjacent sequence.

### Alignment

33,761,231 responder reads and 33,965,033 non-responder reads aligned back to hg19 (99.2% for both pools), of which 31,960,774 and 32,008,412 reads aligned back to the target region, respectively. Of these, 31,082,806 and 31,143,590 reads had unique alignments to the target region (Table S1 in Additional file 1).

The median coverage across the target region was 19,200 for responders (range 0-171,197) and 20,220 for non-responders (0-177,376). 99.28% and 99.10% of targeted bases had coverage ≥ 4 for R and NR, respectively. Figure S1 in Additional file 2 shows the number of reads uniquely mapped to each base of the target region. We see spikes of very high coverage that correspond to amplicon ends overrepresented in sequencing [22].

Coverage was found to be relatively low for one of the two pools for two of the 23 amplicons. The responders have very low coverage for the twentieth amplicon (chr19:39,789,495 to 39,794,767) while the non-responders have very low coverage for the first amplicon (chr19:39,709,955 to 39,714,897) (Figures S1 in Additional file 2 and Figure S2 in Additional file 3). Inspection of the relevant PCR products showed only faint bands. Thus 2/46 amplicon pools are likely to have suffered PCR failures leading to low read counts that were very similar to background coverage or reads that aligned to the rest of the genome that was not targeted via PCR. Fortunately, these amplicons are not located close to *IL28B*.

### Variant calling and association testing

CRISP detected 1221 SNVs and 118 indels (1339 variants in total).

### Validation with original GWAS SNP data and HapMap data

CRISP called all 15 of the SNPs individually typed as part of the GWAS as variants. The Spearman correlation coefficients of MAF estimates between the pooled MPS and individual genotyped data for these SNPs was 0.99 (R) and 0.95 (NR). Odds ratios were well correlated (ρ = 0.98) while p-values were poorly correlated (ρ = 0.35).

31 of the 35 HapMap SNPs in the target region were called as variants; these had CEU MAFs of 0.013 or higher. The four HapMap SNPs not called as variants had CEU MAFs of 0.004, 0.005, 0.009 and 0.027. Spearman correlation coefficients of CEU MAFs with MAFs estimated from pooled MPS data were 0.84 (R) and 0.80 (NR).

### Individual genotyping results

After taking into account obligate missingness, 86 samples with a genotyping rate below 89.47% were discarded, leaving 819 samples. All 19 SNPs had genotyping rates above 90%, with 17 SNPs having genotyping rates of 99% or higher. SNP rs628973 was discarded due to a suspicious distribution of genotypes (98.25% of genotypes heterozygous, Hardy-Weinberg test for combined sample p = 9.78 × 10^-136^). The other 18 SNPs had Hardy-Weinberg test p-values ranging from 3.09 × 10^-8 ^to 0.78, minor allele frequencies ranging from 0.036 to 0.47, and differential missingness p-values ranging from 0.095 to 1. Hence, we performed association analyses using 819 samples with genotypes for up to 18 SNPs. A meta-analysis of allelic test results found that 12 of these SNPs were strongly (p < 0.001) associated with response (Table S2 in Additional file 1). Results under a genotypic genetic model are presented in Table S3 in Additional file 1.

The four SNPs that best tagged the *IL28B *region haplotypes were investigated further for their ability to predict response to therapy. Response prediction was described in terms of a recessive model, given that the response for these SNPs was best described by a recessive model (Table S3 in Additional file 1). The prediction of these four SNPs in their recessive state was also investigated in conjunction with their HLA-C allele, which has also been shown to be important in predicting response to clear the Hepatitis C virus (*PLoS Medicine*, accepted).

**Figure 3:**
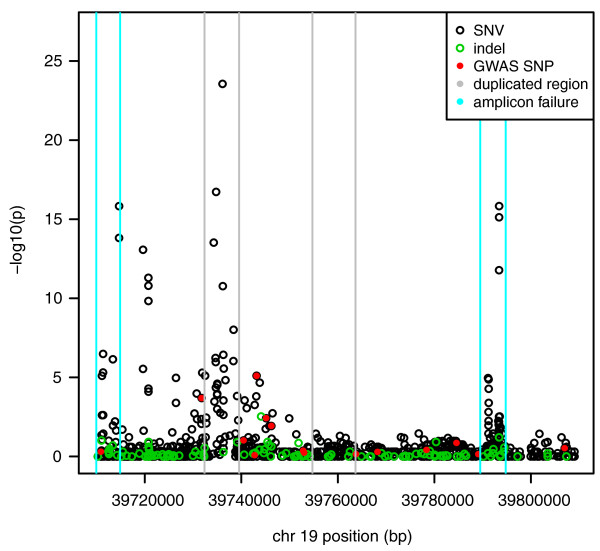
**Results of allele-based association tests at the locations of variants called by CRISP using pooled MPS data**.

The association testing results using Fisher's exact test based on the BWA alignment and CRISP variant calls from the MPS data with rescaled read counts show a strong clustering of association signals around *IL28B*. Both problem amplicons where PCR failures were suspected were retained in the analysis. Figure 3 shows that both of these regions produced spurious association signals with amplicon 1 producing the most significant association signals overall.

**Figure 4:**
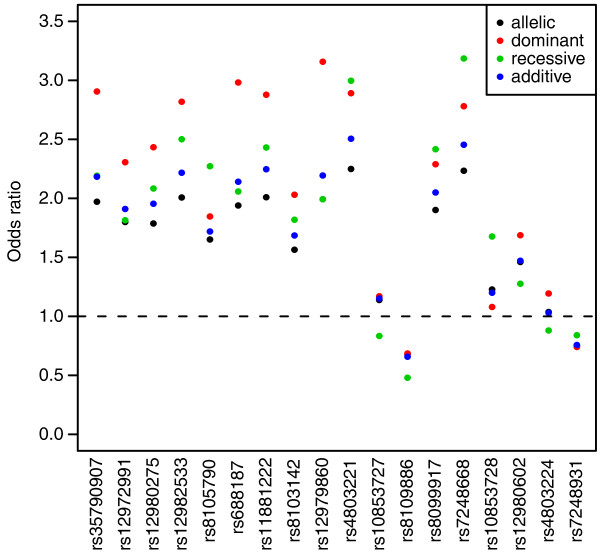
**Odds ratios for the eighteen individually genotyped SNPs under four different genetic models**.

The association testing of the nineteen SNPs chosen for follow up individual genotyping in the independent combined cohort validate the MPS association testing results for the *IL28B *region, previous GWAS results and other more recent re-sequencing results which are not MPS derived, clearly implicating *IL28B *as the most likely causal gene. SNPs rs4803221 and rs7248668 had the highest association for a recessive model with an OR = 4.74 and 4.85 respectively (Figure 4, Table S3 in Additional file 1). Surrounding SNPs closest to this SNP also show clear recessive effects. These results in combination are explained by the haplotype effect displayed in Table 2.

**Table 2:** Table of haplotypes with odds ratios

NUMBER	HAPLOTYPE	HAPLOTYPE TAGGING SNP^A^	FREQUENCY (%)	RESPONDERS (%)	NON-RESPONDERS (%)	*P*-VALUE	OR^B ^(95% CI)
1	ATTGAT**CC**TC**TG**	rs8109886	43.2	48.4	39.6	4.00 × 10^-4^	0.70 (0.57-0.85)
2	GCCAGC**TG**TA**GA**	rs8099917/rs7248668	23.8	16.5	30.4	9.51 × 10^-11^	2.20 (1.72-2.80)
3	GCTAGC**TC**CA**TG**	rs10853727	10.3	10.1	10.6	0.77	1.04 (0.75-1.43)
4	ATTGAT**CC**TA**TG**		9.8	12.3	7.8	2.40 × 10^-3^	0.60 (0.43-0.83)
5	ATTAAC**TC**TA**TG**		1.9	2.2	1.7	0.47	0.79 (0.39-1.60)
6	GCCAGC**TG**TA**TG**		1.4	1.1	1.6	0.39	1.49 (0.62-3.56)

The distribution of the six haplotypes in haplotype block 2 (Fig 4) bound by SNPs rs12980275 and rs7248668. The order is: rs12980275, rs12982533, rs8105790, rs688187, rs11881222, rs8103142, **rs12979860, rs48031221**, rs10853727, rs8109886, **rs8099917, rs7248668**. ^a^SNP in the haplotype block 2 that tag the respective haplotypes. ^b^ORs have been calculated as carriage of the haplotype vs non-carriage of the haplotype.

### Haplotypes and linkage disequilibrium

**Figure 5:**
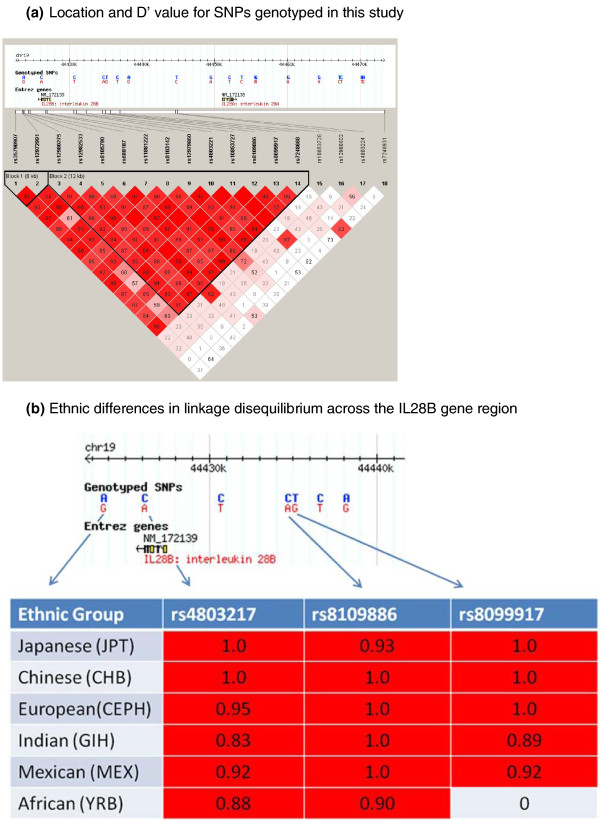
**IL28B Haplotype Blocks**. **(a) **Location and D' values for the SNPs genotyped in this study. Linkage disequilibrium blocks determined from our cohort data using Haploview. HapMap SNPs genotyped in multiple populations shown in the header map in each case. **(b) **Ethnic differences in linkage disequilibrium across the IL28B gene region. r^2 ^values are for the currently available SNPs genotyped in different ethnic groups, with the designated SNPs compared to rs12980275.

In our total cohort, six haplotypes were identified using Haploview (Table 2 Figure 5a). Haplotype 2 had the most significant association with failure to respond to therapy. This was tagged by rs8099917 and rs7248668, and largely by rs4803221. These are also the SNPs with the highest odds ratios for homozygotes.

### Predictive value for failure to respond

To evaluate the utility of the SNPs to predict treatment failure we have compared the sensitivity, specificity, positive (for treatment failure, PPV) and negative predictive values for the SNP minor allele homozygotes. The best four are shown in Table 3. The PPV for treatment failure is likely to be the most useful parameter clinically (see discussion). The SNPs rs4803221 GG (PPV of 77.1, CI 62.7-88.0); rs7248668 AA (PPV 78.3, CI 63.6-89.1) both perform better than the SNPs currently used in testing rs8099917 GG (PPV 73.3, CI 58.1-85.4) and rs12979860 TT (PPV 68.3; CI 59.2-76.5) in this sample. However, the confidence intervals indicate that they may perform better, worse or equivalently at a population level.

**Table 3:** Prediction of failure to clear of virus on therapy with PegIFN/R with homozygote non-responder SNPs (based on 404 responders and 464 non responders of European origin)

Genotype	Sensitivity (95% CI)	Specificity (95% CI)	Positive predictive value (95% CI)	Negative predictive value (95% CI)
rs4803221 GG	8.4 (6.0-11.4)	97.0 (94.7-98.5)	77.1 (62.7-88.0)	47.1 (43.5-50.7)
rs7248668 AA	8.1 (5.7-11.0)	97.3 (95.1-98.7)	78.3 (63.6-89.1)	47.0 (43.4-50.5)
rs8099917 GG	7.4 (5.2-10.3)	96.8 94.5-98.3)	73.3 (58.1-85.4)	46.8 (43.2-50.4)
rs12979860 TT	18.9 (15.4-23.0)	89.5 (85.9-92.5)	68.3 (59.2-76.5)	48.0 (44.2-51.8)

We have recently identified that combination of carrier status for the rs8099917 minor allele with HLA-C C2C2 homozygosity predicts treatment failure (PPV of 80.3) better than either genotype alone (*PLoS Medicine*, accepted), and more people have this genotype than are homozygous for the minor alleles. Here all the haplotype 2 SNPs (rs4803221 G, rs7248668 A, and rs8099917 G) perform similarly (PPV 78.6, 79.7, 80.3), and better than rs12979860 T (PPV 73.1) (Table S4 in Additional file 1). The maximum proportion of the population identified as having a non-responder genotype is by including those with minor allele homozygosity (rs4803221 GG, PPV 77.1) with those who are carriers of rs4803221 G and are HLA-C C2 homozygotes (PPV 78.6). This is 7.7% of the responder cohort, and 19.9% of the non-responders (Table S5 in Additional file 1). Although SNP rs1297860 T homozygosity plus T carriers with HLA-C C2 homozygosity predicts a higher proportion of people who will fail to respond (33.2% of non-responders), it mis-classifies more responders (17.1% responders).

### In silico analysis of transcription factor binding sites and methylation sites in the proximal promoter region

**Figure 6:**
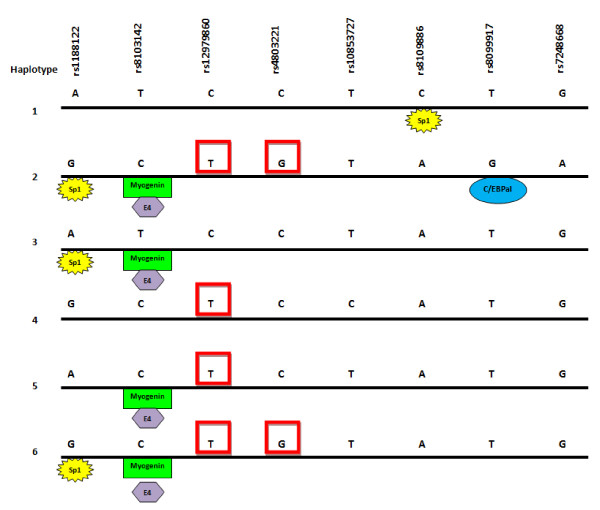
**Putative transcription factor and methylation sites on IL28B haplotypes**. The 6 haplotypes identified using Haploview are shown. SNPs changing CpG sites in the region identified as methylated by the Miklem and Hillier method (unpublished, UCSC Draft Human Genome) are boxed in red. Predicted transcription factor binding sites different between haplotypes were identified using Ali Baba [29]. Note these recognition site differences are from *in silico *analyses only, and serve as a proof of principle that the haplotype sequence differences are sufficient to alter response to transcription factors.

A CpG island encompassing SNPs rs12978960 and rs4803221has been identified on the UCSC human genome draft by Miliak and Hillier (Figure 6),. The major allele for each SNP comprises the C of a CpG dinucleotide. Transcription factors that might differentiate between the haplotypes were sought using the program Ali Baba (Figure 6), which identifies transcription factor sites based on core recognition motifs. Several were identified which varied according to SNP present on the haplotype.

## Discussion

After the discovery that rs8099917 and rs12978960 predicted PegIFN/R response in hepatitis C treatment, much effort has gone into establishing which of these is more likely to be causal, or to be the most useful in a diagnostic test [23,24]. We identified eighteen SNPs in the putative promoter region of *IL28B *using massively parallel sequencing on pooled responders and non-responders, which predicted response to PegIFN/R. These eighteen SNPs collapse to six haplotypes that predict response according to tagging by their minor alleles. The minor allele for SNPs rs8099917, rs7248668, and rs4803221 were all found on one haplotype, haplotype 2, which had the highest OR (recessive model) for predicting treatment failure. The minor allele of SNP rs4803221 is also found on the rare haplotype 6. Minor alleles from SNPs rs12979860, rs11881222, rs688187, rs12982533, rs35790907, rs12982533, rs12980275 were found on this haplotype and two to three others (with MAF > 1%).

The LD block varies between ethnic groups. Japanese and Chinese have fewer common haplotypes, with virtually complete linkage between rs8099917, rs12978960, and the 3' end of the gene. In this population any of the SNPs predict treatment failure with similar precision. People of European descent, Hispanics and Indians have more common haplotypes. In the former two we know that haplotype 2 best predicts failure to respond, with homozygotes for haplotype 2 best predicting treatment failure. There is some recombination between the SNPs tagging haplotype 2, and then the best two are rs7248668 (next to rs8099917) and rs4803221 (next to rs12979860, also on the rare hapotype 6). The African population has the shortest LD block, with a boundary between rs12979860 and rs8099917, and previous work has shown that the major effect of this African haplotype lies between this point and the 3' end of the gene (Ge et al, 2009). The causative SNPs or SNP, or other genetic variant, is therefore most likely to be in this section of the gene, which includes promoter variants, intronic, codon changing, and 3'untranslated SNPs, any of which could be, or could contribute with others, to the functional effect of the haplotype. However, Ge et al identified an effect of rs8099917 independent of this block in African Americans, indicating multiple, variable, haplotype effects on gene function.

### The causative haplotype

Each of the haplotypes has putative immune transcription factor sites distinguishing it from the others. Notably, the haplotype 2 SNP, rs4803221, is in the CpG island, and removes a CpG site. The SNP rs12978960 is the only other common SNP also in this region, and the variant on haplotype 2 also removes a CpG site. Therefore, two potential methylation sites are missing from haplotype 2, and none or one methylation site from the haplotypes corresponding to response. Methylated DNA is resistant to unfolding and corresponds to reduced expression. We hypothesise that increased methylation leads to reduced expression of IL28B, and the interferon sensitive genes (ISGs) upregulated by it, in the responder haplotype; which is then responsive to interferon alpha stimulation on therapy. Two studies have identified that IL28B non-responders have high ISG expression in infected hepatocytes, and that high ISG levels independently predict poor response to therapy [25]. However we, and others, have found that the non-responder haplotype is associated with reduced expression in peripheral blood of healthy controls [3,4]. Other authors have also found no evidence for an effect of IL28B genotype on ISG expression in uninfected hepatocytes [26]. This suggests expression of IL28B and ISGs are context dependent.

Haplotype 2 is clearly the major causative haplotype. The causative SNP or SNPs will probably best be sought in the African American population, where the linkage disequilibrium is lowest, allowing breaking up of the large haplotype block and thus allowing inference of the relative effects of each SNP. The task of demonstrating the basis for causality, even of haplotypes, is not trivial, and thus far has not been achieved for most of the genetic associations currently identified by GWAS. In the short term, diagnostic tests are likely to be dependent on haplotype tagging SNPs, in combination with other parameters such as IP10, 25 hydroxy vitamin D_3_, and HLA-C genotype. From genotyping, SNP rs4803221 and HLA-C C2C2 provides the best predictive value.

### Clinical utility

Therapy for HCV infection is currently undergoing a radical transformation with the advent of Direct Acting Antivirals (DAAs) such as boceprivir and telaprivir to the PegIFN/R backbone [27]. Cure rates are substantially higher (20-30%), and these drugs have the potential to reduce treatment duration. However, enthusiasm for these regimens is tempered by the substantially lower cure rates (~30%) in previous PegIFN/R null responders. In all those failing single DAA-based therapy, future treatment with multiple DAA-based combinations with or without PegIFN/R may be compromised by the development of drug resistance. Further, HCV eradication using single DAA-based strategies, particularly in previous treatment failures appears to be *IL28B *genotype dependent. In this context, predicting non-response rather than success is paramount since the former should perhaps have therapy deferred until multiple DAA-based combinations become available. The present results therefore provide a strong rationale for the use of rs4803221 in combination with the HLA-C genotype, such that those with non-response genotypes be considered for future regimens rather than single DAA-based therapy, signalling the emergence of truly personalized treatment for HCV infection.

### Resequencing for GWAS with MPS

Due to cost constraints the current approaches for GWAS re-sequencing usually employ pooling strategies. Large pool sizes make it difficult to differentiate between rare variants and sequence errors, while the variable representation of each haplotype among those sequenced makes MAF estimates more variable and complicates association testing. However, if analysis is performed carefully it is possible to not just identify variants but to also meaningfully assess the association signals. This is the first time that this region has been re-sequenced with MPS to discover novel variants that could determine the response to hepatitis C treatment. No rare variants tagging haplotype 2 were identified, but this does not exclude the existence of a synthetic association via multiple rare variants. Such rare variants would have to be predominantly on haplotype 2.

MPS resequencing in GWAS is not without problems. Targeting of the region is required, usually with PCR, for smaller association signals. PCR can lead to amplification artefacts that can be alleviated, at least in part, with advanced lab protocols [22] which would result in more uniform coverage than what was achieved in this study. Failure to assess amplicon pool performance can clearly lead to false positive association signals in MPS resequencing studies. Genomic regions with high homology, such as the one that *IL28B *is located in, have low mapability, resulting in reduced ability to detect new variants. Another problem is that short reads can misalign to a different version of the homologous sequence than the one they were derived from, resulting in alignments with mismatches that are incorrectly identified as putative variants. Using an alignment program such as BWA, which allows for gapped alignment around indels, is crucial. Thus this study has provided important insights for future resequencing studies following up GWAS hits.

Although we found no evidence for SNPs with even bigger odds ratios than the haplotype taggers, rare variants (< 5%) would not have been detected in this study, since with the large pool sizes used the lowest theoretical MAF would be similar in magnitude to the frequency of sequencing errors. To improve sensitivity for such detection, smaller pool sizes could be used or samples sequenced individually (e.g. using barcoding); these options become increasingly feasible as the cost of MPS decreases. Newer sequencing technologies generate longer reads, which will allow more read pairs to be aligned uniquely to this highly repetitive region, resulting in higher coverage and more power to detect variants. Protocols to reduce PCR amplification artefacts will also be refined so that MPS of PCR products can be more effectively performed, resulting in more even coverage and thus better ability to detect variants.

## Conclusions

Pooled massive parallel sequencing followed by individual genotyping in a large cross sectional cohort has allowed identification of the common SNPs around the IL28B gene which best predicted response to PegIFN/R for the treatment of hepatitis C. We argue that prediction of failure to respond to therapy is the most useful parameter for clinical management. SNPs tagging haplotype 2 provide the best positive predictive value of treatment failure. This haplotype was conserved from the 3' end of the gene to 8kb upstream of the 5' end of the gene in people of European descent. This causal haplotype contains different putative transcription factor binding sites and two SNPs predicted to abolish the methylation potential of CpG sites located within a CpG island. These data are consistent with the haplotypes altering drug response through regulation of expression of IL28B.

## Abbreviations

GWAS: genome wide association study; HCV: hepatitis C virus; ISG: interferon sensitive gene; LD: linkage disequilibrium; MAF: minor allele frequency; MPS: massively parallel sequencing; OR: odds ratio; PCR: polymerase chain reaction; PegIFN/R: pegylated interferon alpha and ribavirin; PPV: positive predictive value; SNP: single nucleotide polymorphism; SNV: single nucleotide variant.

## Competing interests

Authors KRS, VS, GS, MB, JG and DB have patent applications for the use of IL28B SNPs for diagnostic purposes in immune diseases. The other authors declare that they have no competing interests.

## Authors' contributions

KS analysed the MPS data, performed additional statistical analysis and co-wrote the manuscript. VS designed and performed the PCR reactions, analysed data and co-wrote the paper. MB advised and contributed to the statistical analysis of the data and co-wrote the manuscript. DB conceptualized and designed the experiment, contributed to the analysis and co-wrote the paper. All other authors contributed samples for the study, and/or contributed to the writing of the paper.

## Supplementary Material

Additional file 1 **Tables S1 to S5**. Table S1: number of reads aligned for each comparison. Table S2: results of the allelic test for 18 individually genotyped SNPs. Table S3: results of the genotypic test for 18 individually genotyped SNPs. Table S4: prediction of failure to clear virus on therapy with PegIFN/R with HLA-C/IL28B SNP combination. Table S5: HLA-C combination with IL28B SNPs. Click here for file

Additional file 2 **Figure S1**. Coverage of the target region for responder and non-responder pools. Coverage spikes around the locations of primers (indicated by green dotted lines). Click here for file

Additional file 3 **Figure S2**. Coverage of amplicons 1 and 20 for responders (black) and non-responders (red). Click here for file

## References

[B1] Ge D Fellay J Thompson AJ Simon JS Shianna KV Urban TJ Heinzen EL Qiu P Bertelsen AH Muir AJ Sulkowski M McHutchison JG Goldstein DB Ge D Fellay J Thompson AJ Simon JS Shianna KV Urban TJ Heinzen EL Qiu P Bertelsen AH Muir AJ Sulkowski M McHutchison JG Goldstein DB Genetic variation in IL28B predicts hepatitis C treatment-induced viral clearance. Nature 2009 461 399 401 10.1038/nature08309 19684573

[B2] Rauch A Kutalik Z Descombes P Cai T di Iulio J Mueller T Bochud M Battegay M Bernasconi E Borovicka J Colombo S Cerny A Dufour J-F Furrer H G¸nthard HF Heim M Hirschel B Malinverni R Moradpour D M¸llhaupt B Witteck A Beckmann JS Berg T Bergmann S Negro F Telenti A Bochud P-Y Genetic variation in IL28B Is Associated with Chronic Hepatitis C and Treatment Failure - A Genome-Wide Association Study. Gastroenterology 2010 138 1338 1345 e1337 10.1053/j.gastro.2009.12.056 20060832

[B3] Suppiah V Moldovan M Ahlenstiel G Berg T Weltman M Abate ML Bassendine M Spengler U Dore GJ Powell E Riordan S Sheridan D Smedile A Fragomeli V Muller T Bahlo M Stewart GJ Booth DR George J Suppiah V Moldovan M Ahlenstiel G Berg T Weltman M Abate ML Bassendine M Spengler U Dore GJ Powell E Riordan S IL28B is associated with response to chronic hepatitis C interferon-alpha and ribavirin therapy. Nat Genet 2009 41 1100 1104 10.1038/ng.447 19749758

[B4] Tanaka Y Nishida N Sugiyama M Kurosaki M Matsuura K Sakamoto N Nakagawa M Korenaga M Hino K Hige S Ito Y Mita E Tanaka E Mochida S Murawaki Y Honda M Sakai A Hiasa Y Nishiguchi S Koike A Sakaida I Imamura M Ito K Yano K Masaki N Sugauchi F Izumi N Tokunaga K Mizokami M Tanaka Y Genome-wide association of IL28B with response to pegylated interferon-alpha and ribavirin therapy for chronic hepatitis C. Nat Genet 2009 41 1105 1109 10.1038/ng.449 19749757

[B5] Thomas DL Thio CL Martin MP Qi Y Ge D O'hUigin C Kidd J Kidd K Khakoo SI Alexander G Goedert JJ Kirk GD Donfield SM Rosen HR Tobler LH Busch MP McHutchison JG Goldstein DB Carrington M Genetic variation in IL28B and spontaneous clearance of hepatitis C virus. Nature 2009 461 798 801 10.1038/nature08463 19759533 PMC3172006

[B6] Lagging M Askarieh G Negro F Bibert S Söderholm J Westin J Lindh M Romero A Missale G Ferrari C Neumann AU Pawlotsky J-M Haagmans BL Zeuzem S Bochud P-Y Hellstrand K for the D-HCVSG Response Prediction in Chronic Hepatitis C by Assessment of IP-10 and *IL28B*-Related Single Nucleotide Polymorphisms. PLoS ONE 2011 6 e17232 10.1371/journal.pone.0017232 21390311 PMC3044738

[B7] Bitetto D Fattovich G Fabris C Ceriani E Falleti E Fornasiere E Pasino M Ieluzzi D Cussigh A Cmet S Pirisi M Toniutto P Complementary role of vitamin D deficiency and the interleukin-28B rs12979860 C/T polymorphism in predicting antiviral response in chronic hepatitis C. Hepatology 2011 53 1118 1126 10.1002/hep.24201 21480318

[B8] Urban TJ Thompson AJ Bradrick SS Fellay J Schuppan D Cronin KD Hong L McKenzie A Patel K Shianna KV McHutchison JG Goldstein DB Afdhal N IL28B genotype is associated with differential expression of intrahepatic interferon-stimulated genes in patients with chronic hepatitis C. Hepatology 2010 52 1888 1896 10.1002/hep.23912 20931559 PMC3653303

[B9] Suppiah V Gaudieri S Armstrong N O'Connor KS Berg T Weltman M Abate ML Spengler U Bassendine M Dore GJ Irving WL Powell E Hellard M Riordan S Mathews G Sheridan D Nattermann J Smedile A Müller T Hammond E Dunn D Negro F Bochud P-Y Mallal S Ahlenstiel G Stewart GJ George J Booth DR the International Hepatitis C Genetics Consortium (IHCGC) IL28B, HLA-C and KIR variants additively and interactively predict response to therapy in chronic hepatitis C virus infection. PLoS Med 2011 8 e1001092 10.1371/journal.pmed.1001092 21931540 21931540 PMC3172251

[B10] di Iulio J Ciuffi A Fitzmaurice K Kelleher D Rotger M Fellay J Martinez R Pulit S Furrer H Günthard HF Battegay M Bernasconi E Schmid P Hirschel B Barnes E Klenerman P Telenti A Rauch A the Swiss HIVCS Estimating the net contribution of interleukin-28B variation to spontaneous hepatitis C virus clearance. Hepatology 2011 53 1446 1454 10.1002/hep.24263 21360716 PMC3128709

[B11] Ito K Higami K Masaki N Sugiyama M Mukaide M Saito H Aoki Y Sato Y Imamura M Murata K The rs8099917 Polymorphism, Determined by a Suitable Genotyping Method, is a Better Predictor for Response to Pegylated Interferon-{alpha}/Ribavirin Therapy in Japanese Patients than Other SNPs Associated with IL28B. J Clin Microbiol 2011 49 1853 1860 10.1128/JCM.02139-10 21389156 PMC3122695

[B12] Rhead B Karolchik D Kuhn RM Hinrichs AS Zweig AS Fujita PA Diekhans M Smith KE Rosenbloom KR Raney BJ Pohl A Pheasant M Meyer LR Learned K Hsu F Hillman-Jackson J Harte RA Giardine B Dreszer TR Clawson H Barber GP Haussler D Kent WJ The UCSC Genome Browser database: update 2010. Nucleic Acids Research 2010 38 D613 D619 10.1093/nar/gkp939 19906737 PMC2808870

[B13] Sheppard P Kindsvogel W Xu W Henderson K Schlutsmeyer S Whitmore TE Kuestner R Garrigues U Birks C Roraback J Ostrander C Dong D Shin J Presnell S Fox B Haldeman B Cooper E Taft D Gilbert T Grant FJ Tackett M Krivan W McKnight G Clegg C Foster D Klucher KM IL-28, IL-29 and their class II cytokine receptor IL-28R. Nat Immunol 2003 4 63 68 10.1038/ni873 12469119

[B14] Andrews S: FASTQC. A quality control tool for high throughput sequence data. http://www.bioinformatics.bbsrc.ac.uk/projects/fastqc/

[B15] The European Genome-phenome Archive (EGA). http://www.ebi.ac.uk/ega/

[B16] Li H Durbin R Li H Durbin R Fast and accurate short read alignment with Burrows-Wheeler transform. Bioinformatics 2009 25 1754 1760 10.1093/bioinformatics/btp324 19451168 PMC2705234

[B17] Li H Handsaker B Wysoker A Fennell T Ruan J Homer N Marth G Abecasis G Durbin R Genome Project Data Processing S Li H Handsaker B Wysoker A Fennell T Ruan J Homer N Marth G Abecasis G Durbin R The Sequence Alignment/Map format and SAMtools. Bioinformatics 2009 25 2078 2079 10.1093/bioinformatics/btp352 19505943 PMC2723002

[B18] Bansal V A statistical method for the detection of variants from next-generation resequencing of DNA pools. Bioinformatics 2010 26 i318 324 10.1093/bioinformatics/btq214 20529923 PMC2881398

[B19] The International HapMap Consortium A second generation human haplotype map of over 3.1 million SNPs. Nature 2007 449 851 861 10.1038/nature06258 17943122 PMC2689609

[B20] Purcell S Neale B Todd-Brown K Thomas L Ferreira MA Bender D Maller J Sklar P de Bakker PI Daly MJ Sham PC Purcell S Neale B Todd-Brown K Thomas L Ferreira MAR Bender D Maller J Sklar P de Bakker PIW Daly MJ Sham PC PLINK: a tool set for whole-genome association and population-based linkage analyses. Am J Hum Genet 2007 81 559 575 10.1086/519795 17701901 PMC1950838

[B21] Barrett JC Fry B Maller J Daly MJ Haploview: analysis and visualization of LD and haplotype maps. Bioinformatics 2005 21 263 265 10.1093/bioinformatics/bth457 15297300

[B22] Harismendy O Frazer K Method for improving sequence coverage uniformity of targeted genomic intervals amplified by LR-PCR using Illumina GA sequencing-by-synthesis technology. Biotechniques 2009 46 229 231 10.2144/000113082 19317667

[B23] Afdhal NH McHutchison JG Zeuzem S Mangia A Pawlotsky J-M Murray JS Shianna KV Tanaka Y Thomas DL Booth DR Goldstein DB for the P, Hepatitis CMP Hepatitis C pharmacogenetics: State of the art in 2010. Hepatology 2011 53 336 345 10.1002/hep.24052 21254181

[B24] Ahlenstiel G Booth D George J IL28B in hepatitis C virus infection: translating pharmacogenomics into clinical practice. Journal of Gastroenterology 2010 45 903 910 10.1007/s00535-010-0287-4 20635099

[B25] Honda M Sakai A Yamashita T Nakamoto Y Mizukoshi E Sakai Y Yamashita T Nakamura M Shirasaki T Horimoto K Tanaka Y Tokunaga K Mizokami M Kaneko S Hepatic ISG Expression Is Associated With Genetic Variation in Interleukin 28B and the Outcome of IFN Therapy for Chronic Hepatitis C. Gastroenterology 2010 139 499 509 10.1053/j.gastro.2010.04.049 20434452

[B26] O'Brien TR Interferon-alfa, interferon-lambda and hepatitis C. Nat Genet 2009 41 1048 1050 10.1038/ng.453 19749756 PMC11104492

[B27] Aronsohn A Jensen D Distributive justice and the arrival of direct acting antivirals. Who should be first in line?. Hepatology 2011 53 1789 1791 10.1002/hep.24374 21520197 21520197

[B28] Genome Bioinformatics Group of UC Santa Cruz: Human (*Homo sapiens*) Genome Browser Gateway. http://genome.ucsc.edu

[B29] Grabe N AliBaba 2.1. http://www.gene-regulation.com/pub/programs.html

