# Supplementary material for: Identification of improved IL28B SNPs and haplotypes for prediction of drug response in treatment of hepatitis C using massively parallel sequencing in a cross-sectional European cohort
Source: Genome Med. 2011 Aug 31;3(8):57. doi: 10.1186/gm273 (PMC3238183; doi:10.1186/gm273)
Supplement: Additional file 1 — Tables S1 to S5. Table S1: number of reads aligned for each comparison. Table S2: results of the allelic test for 18 individually genotyped SNPs. Table S3: results of the genotypic test for 18 individually genotyped SNPs. Table S4: prediction of failure to clear virus on therapy with PegIFN/R with HLA-C/IL28B SNP combination. Table S5: HLA-C combination with IL28B SNPs. [file gm273-S1.DOC]

**Table S1. Number of reads aligned for each comparison**

|  | **Responder** | **Non-Responder** |
| --- | --- | --- |
| Reads | 34,026,026 (100.0) | 34,236,380 (100) |
| Aligned to hg19 | 33,761,231 (99.2) | 33,965,033 (99.2) |
| Aligned to target | 31,960,774 (93.9) | 32,008,412 (93.5) |
| Aligned uniquely to target | 31,082,806 (91.4) | 31,143,590 (91.0) |

**Table S2. Results of the allelic test for 18 individually genotyped SNPs. R=responder, NR=nonresponder.**

| **SNP** | **Position** | **Major/minor allele** | **Freq minor allele (NR)** | **Freq minor allele (R)** | **Odds ratio^a^**  **(95% CI)** | **p-value** | **Meta-analysis odds ratio^a^** | **Meta-analysis p-value** |
| --- | --- | --- | --- | --- | --- | --- | --- | --- |
| rs35790907 | 39,730,755 | A/T | 0.478 | 0.317 | 1.97  (1.61,2.41) | 4.08E-11 | 1.98 | 6.06E-11 |
| rs12972991 | 39,731,747 | A/C | 0.370 | 0.246 | 1.80  (1.45,2.23) | 7.51E-08 | 1.80 | 1.20E-07 |
| rs12980275 | 39,731,783 | A/G | 0.471 | 0.332 | 1.79  (1.46,2.19) | 1.42E-08 | 1.79 | 2.25E-08 |
| rs12982533 | 39,731,904 | T/C | 0.482 | 0.317 | 2.01  (1.64,2.46) | 1.30E-11 | 2.01 | 2.33E-11 |
| rs8105790 | 39,732,501 | T/C | 0.330 | 0.230 | 1.65  (1.25,2.18) | 3.71E-04 | 1.63 | 7.00E-04 |
| rs688187 | 39,732,752 | G/A | 0.498 | 0.338 | 1.94  (1.59,2.37) | 7.74E-11 | 1.94 | 1.42E-10 |
| rs11881222 | 39,734,923 | A/G | 0.472 | 0.308 | 2.01  (1.64,2.46) | 1.52E-11 | 2.02 | 2.36E-11 |
| rs8103142 | 39,735,106 | T/C | 0.513 | 0.403 | 1.56  (1.21,2.02) | 5.93E-04 | 1.50 | 2.17E-03 |
| rs12979860 | 39,738,787 | C/T | 0.491 | 0.326 | 1.99  (1.62,2.44) | 3.14E-11 | 1.99 | 6.47E-11 |
| rs4803221 | 39,739,129 | C/G | 0.350 | 0.193 | 2.25  (1.79,2.83) | 2.35E-12 | 2.24 | 9.57E-12 |
| rs10853727 | 39,740,463 | T/C | 0.118 | 0.105 | 1.14  (0.83,1.56) | 0.420 | 1.13 | 0.463 |
| rs8109886 | 39,742,762 | A/C | 0.427 | 0.524 | 0.68  (0.56,0.83) | 1.40E-04 | 0.67 | 1.38E-04 |
| rs8099917 | 39,743,165 | T/G | 0.325 | 0.202 | 1.90  (1.51,2.39) | 2.30E-08 | 1.91 | 4.20E-08 |
| rs7248668 | 39,743,821 | G/A | 0.337 | 0.186 | 2.23  (1.77,2.81) | 5.60E-12 | 2.24 | 1.77E-11 |
| rs10853728 | 39,745,146 | C/G | 0.437 | 0.387 | 1.23  (1.00,1.50) | 0.048 | 1.19 | 0.127 |
| rs12980602 | 39,752,820 | T/C | 0.291 | 0.219 | 1.46  (1.16,1.84) | 0.001 | 1.46 | 0.002 |
| rs4803224 | 39,753,014 | C/G | 0.442 | 0.433 | 1.04  (0.85,1.27) | 0.725 | 0.95 | 0.683 |
| rs7248931 | 39,781,583 | A/G | 0.032 | 0.042 | 0.75  (0.45,1.26) | 0.279 | 0.79 | 0.368 |

^a^ Odds of minor allele among NR divided by odds of minor allele among R

Table S3. Results of the genotypic test for 18 individually genotyped SNPs. R=responder, NR=nonresponder.

| **SNP** | **Position** | **Minor/major** | **NR genotype counts^a^** | **R genotype counts^a^** | **OR1^b^** | **OR2^c^** | **P-value^d^** |
| --- | --- | --- | --- | --- | --- | --- | --- |
| rs35790907 | 39730755 | T/A | 80/265/100 | 34/169/171 | 2.68 | 4.02 | 3.71e-12 |
| rs12972991 | 39731747 | C/A | 45/237/160 | 22/140/212 | 2.24 | 2.71 | 2.92e-08 |
| rs12980275 | 39731783 | G/A | 79/261/105 | 35/178/160 | 2.23 | 3.44 | 5.47e-09 |
| rs12982533 | 39731904 | C/T | 85/259/101 | 32/171/168 | 2.52 | 4.42 | 4.13e-12 |
| rs8105790 | 39732501 | C/T | 25/133/119 | 10/90/139 | 1.73 | 2.92 | 0.0011 |
| rs688187 | 39732752 | A/G | 88/267/90 | 40/173/161 | 2.76 | 3.94 | 4.17e-12 |
| rs11881222 | 39734923 | G/A | 78/263/103 | 30/169/173 | 2.61 | 4.37 | 2.29e-12 |
| rs8103142 | 39735106 | C/T | 58/152/51 | 30/118/73 | 1.84 | 2.77 | 0.00097 |
| rs12979860 | 39738787 | T/C | 82/261/90 | 38/160/164 | 2.97 | 3.93 | 6.82e-13 |
| rs4803221 | 39739129 | G/C | 37/234/169 | 11/121/238 | 2.72 | 4.74 | 6.51e-13 |
| rs10853727 | 39740463 | C/T | 2/97/328 | 2/71/283 | 1.18 | 0.86 | NA |
| rs8109886 | 39742762 | C/A | 65/235/127 | 97/179/80 | 0.83 | 0.42 | 0.00011 |
| rs8099917 | 39743165 | G/T | 33/223/189 | 12/127/235 | 2.18 | 3.42 | 2.24e-08 |
| rs7248668 | 39743821 | A/G | 36/228/181 | 10/118/244 | 2.61 | 4.85 | 3.25e-12 |
| rs10853728 | 39745146 | G/C | 102/169/156 | 56/163/136 | 0.90 | 1.59 | 0.016 |
| rs12980602 | 39752820 | C/T | 30/187/208 | 20/116/220 | 1.71 | 1.59 | 0.0015 |
| rs4803224 | 39753014 | G/C | 98/181/148 | 90/128/138 | 1.32 | 1.02 | 0.19 |
| rs7248931 | 39781583 | G/A | 1/26/417 | 1/29/343 | 0.74 | 0.82 | NA |

^a^ Genotype counts are displayed in the form minor allele homozygote/ heterozygote/ major allele homozygote

^b^ odds of heterozygote vs major homozygous genotype in NR / odds of heterozygote vs major homozygous genotype in R

^c^ odds of minor homozygote vs major homozygous genotype in NR / odds of minor homozygote vs major homozygous genotype in R

^d^ from genotypic chi-square test, i.e. general test of independence between genotypes and response status. NA indicates that a genotypic test was not performed due to at least one genotype count being less than five.

**Table S4. Prediction of failure to clear virus on therapy with PegIFN/R with HLA-C/IL28B SNP combination (based on 404 responders and 464 non responders of European origin).** F- treatment failure, S-Treatment Success

| **Genotype** | **Sensitivity** | **Specificity** | **PPV** | **NPV** |
| --- | --- | --- | --- | --- |
| **rs12979860** |  |  |  |  |
| **F: C2-C2 T*** | **17.5** | **92.3** | **73.1** | **48.3** |
| **S: C1* CC** | 16.8 | 92.2 | 64.2 | 57.1 |
|  |  |  |  |  |
| **rs4803221** |  |  |  |  |
| **F: C2-C2 G*** | **12.6** | **95.9** | **78.6** | **47.8** |
| **S: C1* CC** | 25.3 | 84.9 | 58.2 | 57.6 |
|  |  |  |  |  |
| **rs7248668** |  |  |  |  |
| **F: C2-C2 A*** | **12.4** | **96.2** | **79.7** | **47.6** |
| **S: C1* GG** | 25.3 | 84.7 | 57.8 | 57.8 |
|  |  |  |  |  |
| **rs8099917** |  |  |  |  |
| **F: C2-C2 G*** | **12.2** | **96.4** | **80.3** | **48.2** |
| **S: C1* TT** | 23.8 | 83.1 | 54.4 | 56.3 |

**Table S5**. HLA-C combination with IL28B SNPs

| **HLA-C** | **rs12979860** | **Responders (n=362)** | **Non-responders (n=434)** | **P value** | **OR, 95% CI** |
| --- | --- | --- | --- | --- | --- |
| **C1-C1** | CC | 61 (16.9) | 34 (7.8) |  |  |
|  | CT | 67 (18.5) | 100 (23.0) |  |  |
|  | TT | 10 (2.8) | 33 (7.6) |  |  |
| **C1-C2** | CC | 80 (22.1) | 43 (9.9) |  |  |
|  | CT | 70 (19.3) | 100 (23.0) |  |  |
|  | TT | 24 (6.6) | 35 (8.1) |  |  |
| **C2-C2** | CC | 22 (6.1) | 13 (3.0) |  |  |
|  | CT | 23 (6.4) | 60 (13.8) |  |  |
|  | TT | 5 (1.4) | 16 (3.7) |  |  |
|  |  |  |  |  |  |
| **Rx failure** |  |  |  |  |  |
| **C2-C2 T*** |  | 28 (7.7) | 76 (17.5) | 4.59 x 10^-5^ | 2.5,  1.60-4.00 |
| **others** |  | 334 (92.3) | 358 (82.5) |  |  |
|  |  |  |  |  |  |
| **Rx Success** |  |  |  |  |  |
| **C1* CC** |  | 61 (16.9) | 34 (7.8) | 9.31 x 10^-5^ | 0.42,  0.27-0.65 |
| **others** |  | 301(83.1) | 400(92.2) |  |  |

* rs12982533, rs11881222, rs35790907 have the same distribution as rs12979860.

| **HLA-C** | **rs4803221** | **Responders (n=364)** | **Non-responders (n=436)** | **P value** | **OR, 95% CI** |
| --- | --- | --- | --- | --- | --- |
| **C1-C1** | CC | 92 (25.4) | 66 (15.2) |  |  |
|  | CG | 47 (13.0) | 90 (20.7) |  |  |
|  | GG | 2 (0.6) | 11 (2.5) |  |  |
| **C1-C2** | CC | 106 (29.3) | 73 (16.8) |  |  |
|  | CG | 57 (15.7) | 90 (20.7) |  |  |
|  | GG | 11 (3.0) | 21 (4.8) |  |  |
| **C2-C2** | CC | 34 (9.4) | 30 (6.9) |  |  |
|  | CG | 15 (4.1) | 49 (11.3) |  |  |
|  | GG | 0 | 6 (1.4) |  |  |
|  |  |  |  |  |  |
| **Rx failure** |  |  |  |  |  |
| **C2-C2 G*** |  | 15 (4.1) | 55 (12.6) | 2.29 x 10^-5^ | 3.36,  1.86-6.05 |
| **others** |  | 349 (95.9) | 381 (87.4) |  |  |
|  |  |  |  |  |  |
| **Rx Success** |  | 92 (25.3) | 66 (15.1) | 3.36 x10^-4^ | 0.53,  0.37-0.75 |
| **C1* CC** |  | 272 (74.7) | 370 (84.9) |  |  |
| **others** |  | 92(25.3) | 66(15.1) |  |  |

| **HLA-C** | **rs7248668** | **Responders (n=367)** | **Non-responders (n=443)** | **P value** | **OR, 95% CI** |
| --- | --- | --- | --- | --- | --- |
| **C1-C1** | AA | 2 (0.6) | 12 (2.8) |  |  |
|  | AG | 47 (13.0) | 90 (20.7) |  |  |
|  | GG | 93 (25.7) | 68 (15.7) |  |  |
| **C1-C2** | AA | 10 (2.8) | 19 (4.4) |  |  |
|  | AG | 58 (16.0) | 87 (20.0) |  |  |
|  | GG | 108 (29.8) | 78 (18.0) |  |  |
| **C2-C2** | AA | 0 | 6 (1.4) |  |  |
|  | AG | 14 (3.9) | 49 (11.3) |  |  |
|  | GG | 35 (9.7) | 34 (7.8) |  |  |
|  |  |  |  |  |  |
| **Rx failure** |  |  |  |  |  |
| **C2-C2 A*** |  | 14 (3.8) | 55 (12.4) | 1.27 x 10^-5^ | 3.57,  1.95-6.54 |
| **others** |  | 353 (96.2) | 388 (87.6) |  |  |
|  |  |  |  |  |  |
| **Rx Success** |  |  |  |  |  |
| **C1* GG** |  | 93 (25.3) | 68 (15.3) | 3.9 x 10^-4^ | 0.53,  0.38-0.76 |
| **others** |  | 274 (74.7) | 375 (84.7) |  |  |

| **HLA-C** | **rs8099917** | **Responders (n=366)** | **Non-responders (n=433)** | **P value** | **OR, 95% CI** |
| --- | --- | --- | --- | --- | --- |
| **C1-C1** | GG | 3 (0.8) | 10 (2.3) |  |  |
|  | GT | 50 (13.8) | 82 (18.9) |  |  |
|  | TT | 87 (24.0) | 73 (16.8) |  |  |
| **C1-C2** | GG | 8 (2.2) | 16 (3.7) |  |  |
|  | GT | 64 (17.7) | 85 (19.6) |  |  |
|  | TT | 105 (29.0) | 79 (18.2) |  |  |
| **C2-C2** | GG | 1 (0.3) | 6 (1.4) |  |  |
|  | GT | 12 (3.3) | 47 (10.8) |  |  |
|  | TT | 36 (9.9) | 35 (8.1) |  |  |
|  |  |  |  |  |  |
| **Rx failure** |  |  |  |  |  |
| **C2-C2 A*** |  | 13 (3.6) | 53 (12.2) | 8.78 x 10^-6^ | 3.79,  2.03-7.07 |
| **others** |  | 353 (96.4) | 380 (87.8) |  |  |
|  |  |  |  |  |  |
| **Rx Success** |  |  |  |  |  |
| **C1* GG** |  | 87 (23.8) | 73 (16.9) | 0.015 | 0.65,  0.46-0.92 |
| **others** |  | 279 (76.2) | 360 (83.1) |  |  |
