# Supplementary figures and images for: Identification of improved IL28B SNPs and haplotypes for prediction of drug response in treatment of hepatitis C using massively parallel sequencing in a cross-sectional European cohort
Source: Genome Med. 2011 Aug 31;3(8):57. doi: 10.1186/gm273 (PMC3238183; doi:10.1186/gm273)

**Responders**

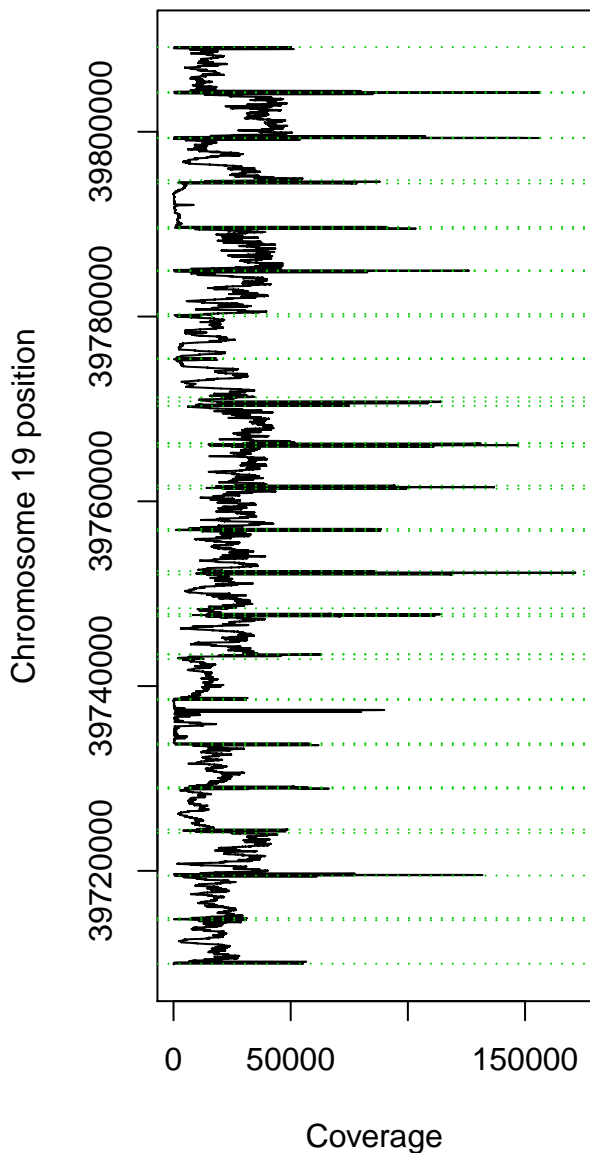

**Nonresponders**

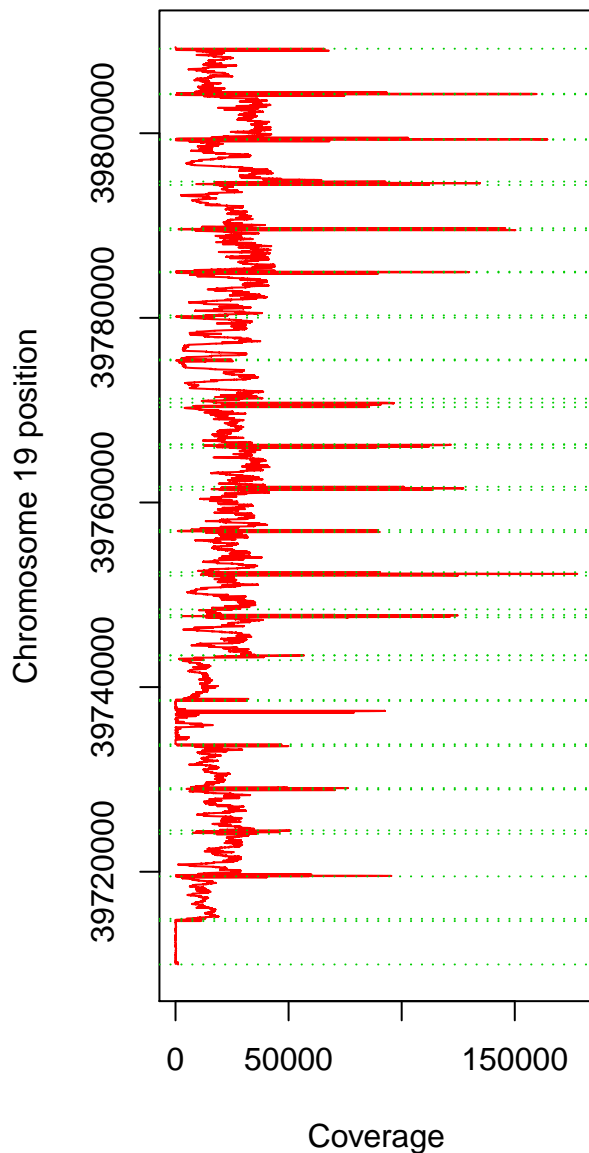

Supplement: Additional file 2 — Figure S1. Coverage of the target region for responder and non-responder pools. Coverage spikes around the locations of primers (indicated by green dotted lines). [file gm273-S2.PDF]

**Amplicon 1**

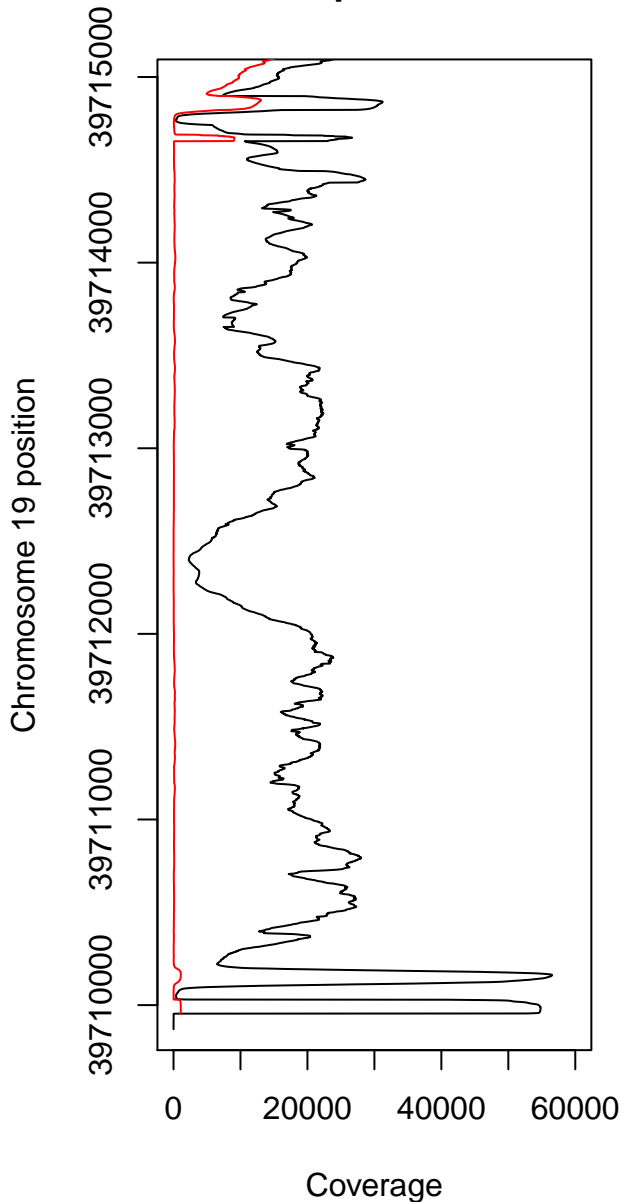

**Amplicon 20**

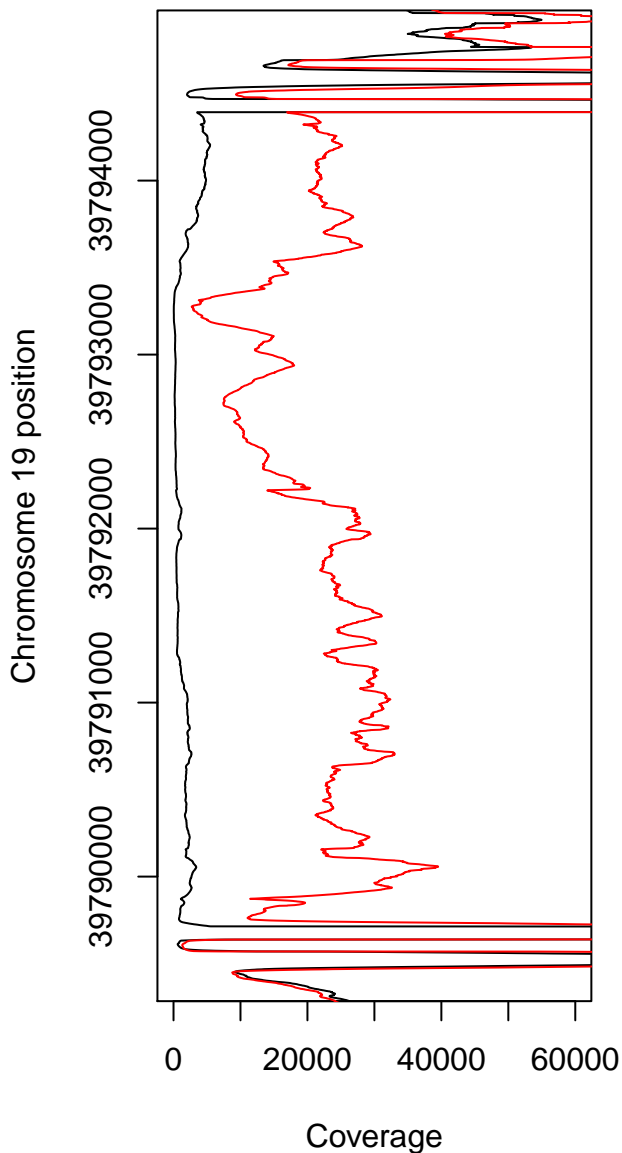

Supplement: Additional file 3 — Figure S2. Coverage of amplicons 1 and 20 for responders (black) and non-responders (red). [file gm273-S3.PDF]
